# 

**Published:** 1886-05

**Authors:** 


### CONTENTS:

Article I—The Occasional Serious Results of Pulp Treatment. By F. M. Dixon, D. D. S ........ 1

Art. II—Principles and Methods of Filling Teeth with Gold. By A G. Bennett, D. D. S ........ 12

Art. III—Diagnosis—Its Importance ........ 21

Art. IV—New Remedies. By C J. Boyd Wallis, L. D. S ........ 23

Art. V—Cocaine in Tooth Extraction. By the Editor of London *Dental Record* ........ 31

Art. VI—Food-Accessories; their Influence on Digestion ........ 37

### EDITORIAL, ETC.

A Defense of English Dentistry ........ 42

American Dentists Abroad ........ 43

### MONTHLY SUMMARY.

Oxyphosphates and Temperature ........ 44

Discoloration of Gold Fillings ........ 44

Danger from the Bicycle ........ 45

White of Egg in Obstinate Diarrhœa ........ 46

A Doctor's Luck ........ 47

Artificial Ivory ........ 47

Repairing a Cracked Plate ........ 47

Sharpening Corundum Wheels ........ 48

Gold Solder ........ 48

